# Recent Advances in the Mechanisms and Applications of *Astragalus* Polysaccharides in Liver Cancer Treatment: An Overview

**DOI:** 10.3390/molecules30132792

**Published:** 2025-06-28

**Authors:** Wang Wang, Hanting Zhou, Akanksha Sen, Pengxia Zhang, Linhong Yuan, Shaobo Zhou

**Affiliations:** 1 Medical College of Basic Sciences, Jiamusi University, Jiamusi 154000, China; wang13836405335@163.com; 2 Key Laboratory of Microecology-Immune Regulatory Network and Related Diseases of Heilongjiang Province, Jiamusi University, Jiamusi 154000, China; 3 School of Medicine, The Chinese University of Hong Kong, Shenzhen (CUHK-Shenzhen), Shenzhen 518172, China; hantingzhou@link.cuhk.edu.cn; 4 School of Science, Faculty of Engineering and Science, University of Greenwich, Medway Campus Central Avenue, Chatham Maritime, Kent ME4 4TB, UK; akanksha.sen@greenwich.ac.uk; 5 School of Public Health, Capital Medical University, Beijing 100069, China

**Keywords:** *Astragalus* polysaccharides, liver cancer, traditional Chinese medicine, immunomodulation, apoptosis, signalling pathways, PI3K/AKT/mTOR, anti-liver cancer mechanisms, applications of *Astragalus* polysaccharides

## Abstract

*Astragalus* polysaccharides (APS), bioactive compounds derived from *Astragalus membranaceus*, have emerged as promising natural agents in the treatment of hepatocellular carcinoma, a leading cause of cancer-related mortality. Preclinical studies indicate that APS exerts significant anti-liver cancer effects through multiple biological actions, including the promotion of apoptosis, inhibition of proliferation, suppression of epithelial–mesenchymal transition, regulation of autophagy, and modulation of immune responses. These therapeutic effects are closely associated with the regulation of critical signalling pathways, such as PI3K/AKT/mTOR, Wnt/β-catenin, JAK/STAT, and TGF-β/Smad. APS also reshapes the tumour microenvironment by enhancing macrophage activity, reducing the regulatory T cell function, and improving host immune response. In addition, APS exhibits synergistic effects when combined with conventional chemotherapeutics and interventional treatments such as transarterial chemoembolisation, improving efficacy and reducing toxicity. Despite the robust experimental evidence, limitations such as low bioavailability and a lack of large-scale clinical trials remain challenges for clinical translation. This review summarises the recent advances in understanding the anti-hepatocellular carcinoma activities of APS, their molecular targets and potential applications, aiming to provide a scientific basis for future studies and the development of APS-based therapeutic strategies.

## 1. Introduction

Liver cancer, particularly hepatocellular carcinoma, remains a global health challenge due to its high incidence, poor prognosis, and limited effectiveness of the current treatment modalities. Traditional Chinese medicine (TCM), widely practiced for centuries, has increasingly gained attention in the oncology field for its holistic and multifaceted approach to cancer management.

Among cancers, liver cancer is one of the most common malignancies worldwide and poses a significant threat to global public health. According to the World Health Organization’s Global Cancer Statistics 2022, approximately 905,000 new cases and 830,000 deaths from liver cancer were reported globally in 2020. Asia, especially China, accounts for a disproportionate share of this burden, driven by the high prevalence of hepatitis infections, dietary risk factors, and cirrhosis [1,2]. With demographic aging and lifestyle changes, the incidence is projected to rise to 1.392 million new cases by 2040, predominantly impacting older individuals in both low- and high-Human-Development Index regions [3]. Despite progress in surgical, chemotherapeutic, immunotherapeutic, and targeted strategies, treatment efficacy remains limited due to late-stage diagnoses and high recurrence rates. These limitations underscore the need for adjunctive or alternative treatments. TCM offers promising prospects by improving clinical outcomes, reducing adverse drug reactions, and enhancing patient quality of life [4,5].

*Astragalus membranaceus* (Fisch.) Bunge is a traditional Chinese medicinal herb, widely known by its Chinese name *Huangqi*. Its dried root, referred to as *Astragali* radix, has gained prominence in cancer therapeutics, primarily due to its bioactive polysaccharide components known as *Astragalus* polysaccharides (APS) [6]. *Astragali* radix, derived from the dried roots of *Astragalus membranaceus* (Fisch.) Bunge and its variety *Mongholicus* (Bunge) Hsiao, contains an extensive array of pharmacologically active constituents that collectively account for its broad therapeutic applications. Contemporary phytochemical investigations have identified over 200 bioactive components, categorised primarily into polysaccharides, triterpenoid saponins, flavonoids, amino acids, trace elements, fatty acids, and phenolic acids [7].

Within the group of these constituents, APS are the most extensively studied due to their potent immunomodulatory and antitumour activities. APS are acidic heteropolysaccharides composed mainly of glucose, galactose, arabinose, and rhamnose [8,9,10]. Their pharmacological efficacy is closely related to their molecular weight, monosaccharide composition, and glycosidic linkage patterns. Notably, alcohol-soluble fractions of APS, enriched in mannose and galactose, display enhanced anti-inflammatory and antioxidant properties [11]. Triterpenoid saponins represent another crucial class of active compounds in *Astragali* radix, particularly astragalosides I–IV and cycloastragenol derivatives. These compounds have shown pronounced cardioprotective, hepatoprotective, neuroprotective, and anti-inflammatory effects [12]. Astragaloside IV is known to modulate calcium homeostasis, inhibit pro-inflammatory signalling pathways such as NF-κB and STAT3, and improve mitochondrial function [13,14]. Flavonoids, including calycosin, formononetin, isorhamnetin, and quercetin, are mainly found in glycosylated and malonylated forms. These polyphenolic compounds contribute significantly to the antioxidant, anti-inflammatory, and oestrogen-like activities of the herb. Their pharmacological efficacy varies with plant maturity, geographical origin, and harvesting season [15,16]. *Astragali* radix also contains a spectrum of amino acids (e.g., proline, glutamic acid, arginine), vitamins (e.g., folic acid), and organic acids (e.g., ferulic acid, caffeic acid), which contribute to nutritional support and immune enhancement [17]. In addition, the herb accumulates trace elements such as selenium, zinc, manganese, and iron—micronutrients that play essential roles in antioxidant defence systems and enzymatic catalysis. Table 1 summarises the major bioactive components of *Astragali* radix (Huangqi), including their representative compounds, and primary biological activities.

Research shows that APS possess diverse pharmacological activities, including immunomodulation, antioxidation, metabolic regulation, and anticancer effects. They enhance immune cell activity, modulate key tumour-related signalling pathways (e.g., PI3K/AKT/mTOR, MAPK, NF-κB), and improve chemotherapy efficacy by sensitising tumour cells and reducing drug resistance and toxicity. APS also contribute to metabolic homeostasis and delay senescence via antioxidant mechanisms. Given these multifunctional properties, this review aimed to summarise the current advances in APS research focused on hepatocellular carcinoma. It highlights the molecular mechanisms underlying APS’s antitumour effects, their impact on the tumour microenvironment, and their potential as adjuncts to conventional therapy, thus supporting future preclinical and clinical translation.

In this review, suitable studies were found using electronic search systems PubMed, Google Scholar, and Scopus. We also searched the bibliographies to identify relevant studies and reviews. The database search was conducted by combining the search terms “APS,” “Liver cancer and Traditional Chinese Medicine,” “Immunomodulation,” “Apoptosis,” “Signalling pathways,” “PI3K/AKT/mTOR,” “anti-liver cancer mechanisms,” and “Applications of APS” with terms such as “Source,” “Chemistry,” “Biological activity,” “Medicinal use,” “Pharmacokinetics,” and “Toxicity profile.”. Based on pharmacological research on APS, as well as in vitro and in vivo experiments, the articles were assessed to determine the most relevant results. Additionally, research and review articles outside the scope of this article were excluded.

## 2. Anti-Liver Cancer Effects of *Astragalus* Polysaccharides (APS)

APS, extracted from *Astragalus membranaceus*, has demonstrated significant anti-hepatocellular carcinoma efficacy in numerous experimental models. Liver cancer remains one of the most lethal malignancies globally, and the search for effective therapeutic agents has led to increased interest in natural compounds such as APS. A robust body of preclinical evidence from both in vitro and in vivo studies supports the anti-liver cancer potential of APS. In murine models, APS administration leads to pronounced tumour suppression. For instance, Lai et al. showed that APS significantly inhibited tumour growth in H22-bearing mice with a dose-dependent effect (100, 200, 400 mg/kg), with the 400 mg/kg group achieving a tumour inhibition rate of 59.01%. Treatments were initiated 24 h after injecting the tumor cells. Drugs were administered once daily for 15 consecutive days [32]. This study provides an initial basis for mechanistic research. While APS may be considered immune-supportive agents for cancer patients, their use must strictly comply with clinical guidelines (250 mg/day) in accordance with the National Cancer Institute’s (NCI) recommendations for managing fatigue and enhancing the immune function. Increasing the dose based on animal studies is strongly discouraged. Future NCI-funded phase III randomised controlled trials are warranted to further evaluate the efficacy and determine the safety window of APS in human cancers. Yang et al. employed an in vivo transplantation tumour model, along with immune organ and cell function assays and toxicity comparisons, to demonstrate that APS enhance the thymus and spleen indices and elevates the key cytokines, including IL-2, IL-12, and TNF-α. These findings further validate the immunomodulatory and antitumour potential of APS [33].

In human liver cancer cell lines, including HepG2 and Hep G2.215, APS show dose-dependent inhibition of cell proliferation and colony formation. Liu et al. reported that APS significantly suppressed the proliferation of HepG2 cells, with the most effective concentration being 200 μg/mL among concentrations of 100, 200, and 400 μg/mL for 5 h [34]. Complementing this, Chan et al. tested the dosage range of 100–1000 μg/mL and demonstrated that even low-dose APS (100 μg/mL) reduced colony formation, with a notable inhibition rate of 39.8% observed at 1000 μg/mL after 48 h [35]. APS also show efficacy in xenograft models and patient-derived cancer cell models. Li et al. demonstrated that APS treatment modulates the tumour microenvironment by reducing the regulatory T cell (Treg) activity and repolarising tumour-associated macrophages (TAMs) from a pro-tumour M2 phenotype to an antitumour M1 phenotype, thereby promoting tumour regression [36].

Moreover, both in vitro and in vivo experimental studies showed that APS enhance the effects of chemotherapeutic agents. When combined with doxorubicin (0–1 µM), APS (0–100 mg/L) improved apoptosis in Hep3B liver cancer cells by reducing O-GlcNAcylation and promoting endoplasmic reticulum stress [37]. Of the tested combinations, 10 mg/L APS combined with 1 µM doxorubicin reduced the cell survival rate to 60% and increased the apoptosis rate by approximately 20–30%. Mechanistically, APS downregulate O-GlcNAc transferase expression and protein stability, while upregulating O-GlcNAcase, thereby reducing O-GlcNAcylation. This modulation intensifies endoplasmic reticulum stress and activates apoptotic pathways, including CHOP. In the BALB/c nude mouse Hep3B xenograft model, intraperitoneal injection of APS (50 mg/kg) combined with doxorubicin (2 mg/kg), administered once every three days for 28 days, resulted in a tumour volume reduction of approximately 30% and a tumour weight reduction of ca. 40% compared to the doxorubicin monotherapy group. Additionally, proteins related to endoplasmic reticulum stress and apoptosis were significantly upregulated in the tumour tissues. In drug-resistant H22 liver cancer cells, APS reversed chemoresistance by downregulating P-glycoprotein and MDR1 mRNA expression, thereby increasing intracellular drug accumulation [38]. In clinical contexts, APS show promise as an adjuvant therapy. In patients undergoing transarterial chemoembolisation (TACE), a standard treatment for intermediate-stage hepatocellular carcinoma, APS are found to reduce serum tumour markers such as alpha-fetoprotein and carbohydrate antigens (e.g., CA199), while also preserving liver function and reducing chemotherapy-induced side effects. These effects contributed to improved treatment tolerance and enhanced quality of life [39].

**Figure 1:**
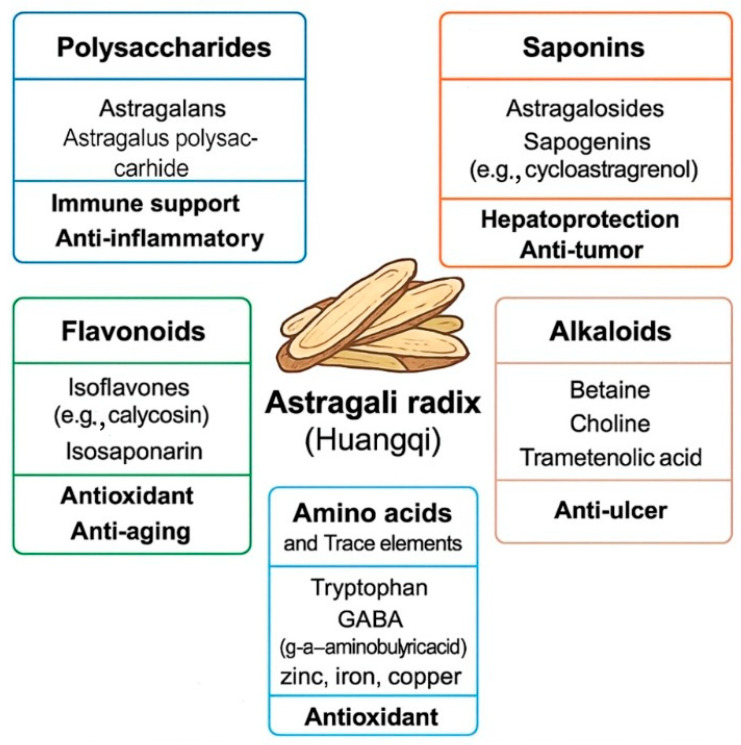
Major bioactive components of *Astragali* radix (Huangqi) and their associated pharmacological functions.

Liver fibrosis is a reparative response of the liver to chronic injury (such as viral hepatitis, alcohol abuse, and fatty liver disease), characterised by excessive deposition of extracellular matrix components. If the condition progresses, it can develop into cirrhosis, which is a major risk factor for hepatocellular carcinoma; notably, 70–90% of hepatocellular carcinoma patients have underlying cirrhosis [40]. Sun et al. combined pharmacological network analysis with animal experiments to investigate the effects of APS on alcoholic liver fibrosis. Initially, 274 common targets of APS were identified through database screening, and a protein–protein interaction network was constructed, highlighting enrichment in the key pathways such as TLR4/JNK/NF-κB. Subsequently, alcoholic liver fibrosis was induced in male Sprague–Dawley rats via intragastric administration of 50% alcohol (8 mL/kg) for 14 days. APS treatment (400 mg/kg/day) demonstrated significant antifibrotic activity. The therapeutic mechanism was closely associated with the downregulation of genes involved in the TLR4/JNK/NF-κB/MyD88 signalling pathway, revealing a novel mechanism and potential therapeutic target for this condition. Importantly, APS treatment was found to inhibit the overexpression of polymerase I and transcript release factor, PTRF/Cavin-1, and reduce the co-localisation of TLR4 with PTRF, thereby alleviating liver damage. The study further indicated that overexpression of PTRF can reverse the hepatoprotective effect of APS in alcoholic liver fibrosis models [41]. *Astragali* radix contains a wide range of active compounds, including polysaccharides (e.g., astragalans and APS), saponins (e.g., astragalosides and cycloastragenol), flavonoids (e.g., calycosin and isosaponarin), alkaloids (e.g., betaine and choline), amino acids, and essential trace elements (e.g., tryptophan, GABA, zinc, iron, and copper); see Figure 1 which summarises these compounds by class, highlighting their representative constituents and associated therapeutic properties.

Taken together, these findings strongly support the anti-liver cancer potential of APS across a wide range of models. Their ability to inhibit tumour growth, modulate immune responses, reverse chemoresistance, and synergise with standard therapies positions APS as promising candidates for integrated liver cancer treatment strategies.

## 3. Mechanisms of Action: APS Exert Their Effects Through Multiple Pathways

### 3.1. Induction of Apoptosis

Apoptosis refers to an orderly, genetically controlled process of autonomous cell death that maintains homeostasis in the internal environment. As a common antitumour strategy, the induction of apoptosis primarily involves two major pathways: the intrinsic (mitochondrial) pathway and the extrinsic (death receptor-mediated) pathway [42,43]. APS activate caspase cascades, downregulate B-cell lymphoma-2 (Bcl-2), and disrupts the mitochondrial membrane potential [32]. One of the most prominent antitumour mechanisms of APS is their ability to induce apoptosis in liver cancer cells, as demonstrated in H22 hepatocellular carcinoma-bearing mice treated with a dosage of 400 mg/kg [32]. APS can activate both the intrinsic and extrinsic apoptotic pathways, leading to caspase activation and DNA fragmentation. Mechanistically, APS downregulate antiapoptotic proteins such as Bcl-2 while upregulating proapoptotic proteins such as Bax and cleaved caspase-3. Moreover, APS have been shown to disrupt the mitochondrial membrane potential and promote the release of cytochrome c, thereby enhancing apoptotic cascades in hepatocellular carcinoma models.

The Bcl-2 family of proteins plays a critical role in regulating apoptosis. Overexpression or aberrant activation of Bcl-2 can promote the survival and growth of cancer cells and enhance treatment resistance. Bcl-2-associated X protein (Bax) is a key executor in mitochondrial-mediated cell death. It functions by permeabilising the mitochondrial outer membrane (MOM). Caspases, a group of structurally related cysteine-aspartic proteases, are involved in cellular processes such as differentiation, programmed cell death, proliferation, and inflammation [44,45,46]. Huang et al. [47] found that the mRNA and protein expression levels of Notch1 were significantly elevated in human hepatocellular carcinoma cells compared to normal cells. APS (0.1, 0.5, and 1.0 mg/mL) were shown to reduce both mRNA and protein levels of Notch1 in a concentration-dependent manner. Furthermore, APS downregulated the expression of Bcl-2 while upregulating Bax and activated the caspase cascade by increasing the levels of caspase-3 and caspase-9, thereby inducing apoptosis in liver cancer cells. Similarly, Lai et al. [32] demonstrated that treatment with APS (100, 200, and 400 mg/kg) upregulated the expression of the proapoptotic protein Bax while downregulating the antiapoptotic protein Bcl-2, thereby inducing apoptosis in liver cancer cells. Notably, the group treated with 400 mg/kg APS achieved a tumour inhibition rate of 59.01%, significantly suppressing tumour growth with relatively low toxicity. These findings suggest that APS exert their antitumour effects primarily through the induction of apoptosis and highlight their potential as a promising therapeutic candidate for hepatocellular carcinoma.

### 3.2. Inhibition of Proliferation

APS arrest the cell cycle at the G0/G1 and G2/M phases and suppress cyclin D1/CDK4. APS have been shown to suppress the proliferation of hepatocellular carcinoma cells by interfering with cell cycle progression, particularly at the G0/G1 and S phases. This antiproliferative effect is typically mediated through the downregulation of cyclins and cyclin-dependent kinases (CDKs), along with the upregulation of tumour suppressor proteins, such as p53 and p21. Uncontrolled cellular proliferation is one of the hallmark features of malignant tumours. While normal cells undergo tightly regulated proliferation, tumour cells bypass these regulatory mechanisms, allowing for continuous and unrestrained division that drives tumour formation and progression [48,49].

**Figure 2:**
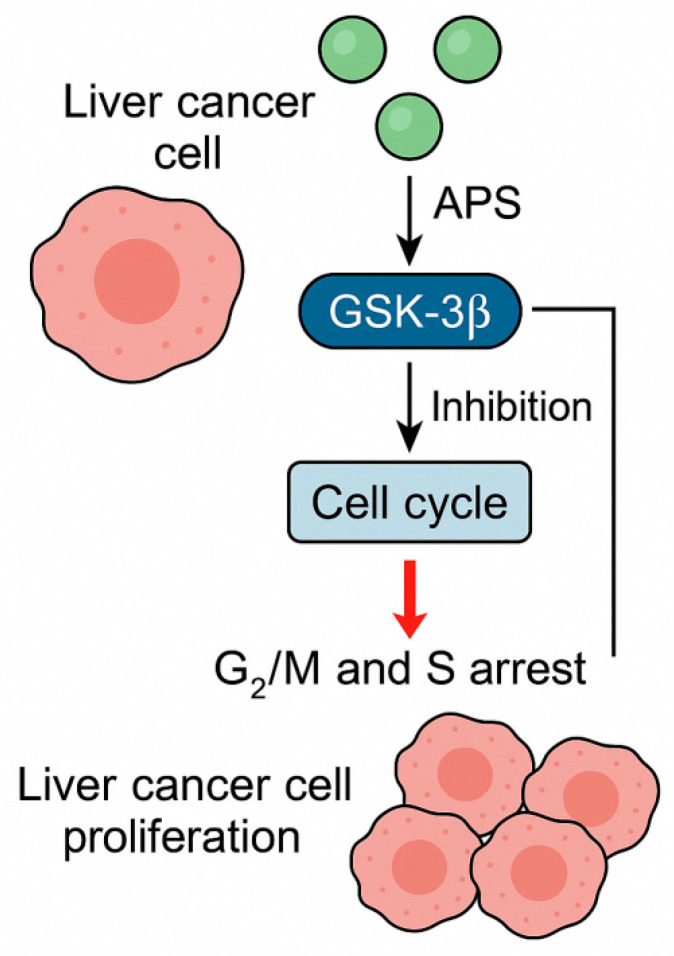
*Astragalus* polysaccharides (APS) inhibit liver cancer cell proliferation by regulating cell cycle arrest through GSK-3β inhibition; the ┐ line means the inhibition of cell proliferation.

In Liu et al.’s [34] study, HepG2 cells were treated with APS (100, 200, and 400 μg/mL) for 5 h, and MTT assay results showed that the absorbance values in all treatment groups from day 1 to day 7 were significantly lower than those of the control group (*p* < 0.05), with the 200 μg/mL group exhibiting the strongest inhibitory effect. Western blot analysis revealed that the expression of the glycogen synthase kinase-3β (GSK3β) protein was significantly reduced in all APS-treated groups compared to the control (*p* < 0.05), and the trend was consistent with the changes observed in absorbance. This demonstrated that APS significantly inhibited the proliferation of HepG2 cells by downregulating the expression of glycogen synthase kinase-3β (GSK-3β), with 200 μg/mL being the most effective concentration. Another study, using a CCK-8 assay, a colony formation assay, and flow cytometry, found that high concentrations of APS (≥800 μg/mL) significantly inhibited cell proliferation, while even low concentrations (100 μg/mL) suppressed colony formation. Flow cytometry results showed a reduction in the proportion of cells in the G2/M phase (10.1% in the control group vs. 2.3–5.5% in the treatment groups) and an increase in the apoptosis rate (7.03% in the control group vs. 9.31–21.89% in the treatment groups). APS markedly suppressed both proliferation and colony formation in HepG2.215 cells. Treatment with 1000 μg/mL of APS for 48 h resulted in a 39.80% inhibition rate, while even a lower concentration of 100 μg/mL significantly reduced colony formation. Mechanistically, APS exerted these effects by inducing cell cycle arrest at the G2/M and S phases, thereby impeding tumour cell proliferation [35]. Given the crucial role of cell cycle regulation in cancer progression, these findings underscore the therapeutic potential of APS as an agent capable of exerting cytostatic effects through modulation of the key cell cycle regulators. These are shown in Figure 2, which shows that APS suppress the expression and activity of glycogen synthase kinase-3β (GSK-3β) in liver cancer cells, leading to the disruption of the cell cycle. This inhibition results in the G2/M and S phase arrest, thereby effectively reducing cancer cell proliferation. The cell cycle blockade induced by APS contributes to its overall antiproliferative effects against hepatocellular carcinoma.

The Wnt/β-catenin signalling pathway plays a critical role in cellular processes such as proliferation, differentiation, migration, and the development and regeneration of tissues and organs. It is also closely associated with the onset and progression of various cancers [50]. APS (both 100 and 200 mg/L) can suppress the activity of the Wnt/β-catenin pathway by downregulating the mRNA and protein expression of β-catenin, C-myc, and cyclin D1, as well as by inhibiting the antiapoptotic gene Bcl-2, thereby inducing apoptosis in HepG2 liver cancer cells [51]. After 72 h of treatment with 400 mg/L APS, the survival rate of HepG2 cells decreased by more than 75%. Zhu et al. [52] reported that treating HepG2 cells with APS (0, 50, 100, and 200 mg/L) for 48 h significantly inhibited cell proliferation in a dose-dependent manner, as shown by the CCK-8 assay (*p* < 0.05). APS also reduced intracellular glutathione (GSH) levels, increased reactive oxygen species (ROS) and lipid peroxidation levels, and elevated intracellular iron ion concentrations—all in a dose-dependent manner.

**Figure 3:**
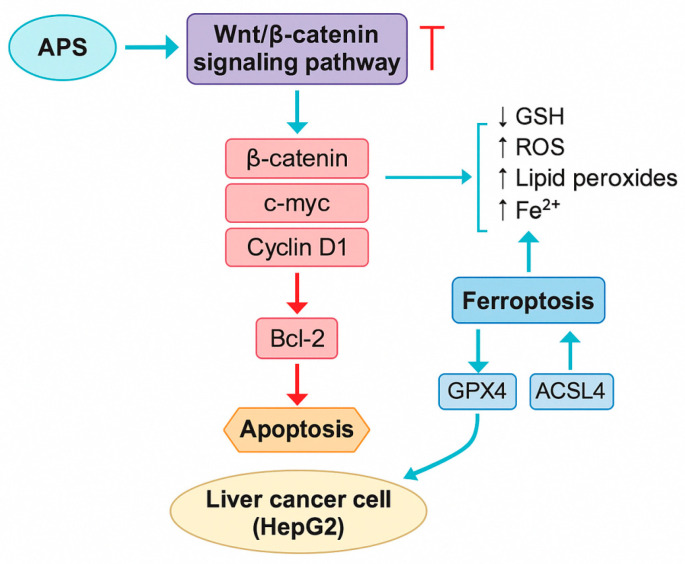
Inhibitory effect of *Astragalus* polysaccharides (APS) on Wnt/β-catenin signalling and induction of apoptosis and ferroptosis in liver cancer cells. The ┬ line means inhibition.

Additionally, APS treatment led to the downregulation of GPX4 and upregulation of ACSL4, indicating that APS promotes ferroptosis in liver cancer cells. Furthermore, APS was shown to inhibit the expression of key proteins involved in the Wnt/β-catenin signalling pathway. These findings suggest that APS may induce apoptosis in liver cancer cells by promoting ferroptosis through the inhibition of the Wnt/β-catenin signalling pathway, providing theoretical support for the prevention and treatment of liver cancer (Figure 3). Figure 3 shows that APS suppress the Wnt/β-catenin signalling pathway by downregulating the key oncogenic targets, including β-catenin, C-myc, and cyclin D1, which subsequently reduces Bcl-2 expression and activates the apoptotic cascade in HepG2 liver cancer cells. Concurrently, APS induce ferroptosis by altering redox homeostasis—marked by reduced glutathione (GSH), increased ROS, elevated lipid peroxidation, and intracellular Fe²^+^ accumulation. This ferroptotic process is mediated through modulation of the ACSL4 and GPX4 activity. The combined activation of apoptosis and ferroptosis contributes to the antitumour effects of APS in hepatocellular carcinoma.

### 3.3. Autophagy Regulation

Autophagy is a lysosome-dependent cellular process that degrades damaged intracellular structures to maintain cellular homeostasis and provide essential substrates for protein synthesis and energy production. It is considered a form of programmed cell death. Dysregulation of autophagy has been associated with tumour development and can interfere with tumour cell apoptosis, angiogenesis, and anticancer treatments [53,54]. Treatment with APS (25 μg/mL) combined with 10 μmol/L of the Akt inhibitor LY294002 for 72 h reduced the survival rate of HepG2 cells to 13.4%, suggesting that APS may regulate autophagy in liver cancer cells by modulating autophagy-related genes and the key signalling pathways. APS inhibited cell viability in a dose-dependent manner, induced G1 phase arrest and apoptosis (with a maximum apoptosis rate of 41.92%), and promoted autophagy by upregulating LC3B while downregulating LC3A and P62. It also inhibited the Akt/p-Akt signalling pathway. When combined with LY294002, apoptosis was further enhanced (apoptosis rate of 58.16%), indicating that APS suppressed hepatoma cell proliferation through dual mechanisms: activation of autophagy and inhibition of the Akt pathway. These findings suggest a novel therapeutic target for liver cancer treatment. Specifically, APS have been shown to upregulate the expression of autophagy-related proteins such as LC3B, Beclin-1, and Atg5, thereby promoting the formation and maturation of autophagosomes. Concurrently, APS inhibit the PI3K/AKT/mTOR signalling pathway, which is a central negative regulator of autophagy. This suppression leads to enhanced autophagic activity, which in some contexts contributes to autophagic cell death.

Evidence suggests that APS may regulate autophagy in a dual-directional manner—by either inducing protective autophagy or promoting excessive autophagic flux that results in cell death, depending on the cellular context and dosage. Such regulation not only contributes to the suppression of liver cancer cell proliferation, but also represents a potential mechanism by which APS enhance the efficacy of anticancer treatments. Through fine-tuning autophagy, APS may thus play a vital role in the broader antitumour response against hepatocellular carcinoma.

#### 3.3.1. Regulation of Autophagy-Related Proteins

**Figure 4:**
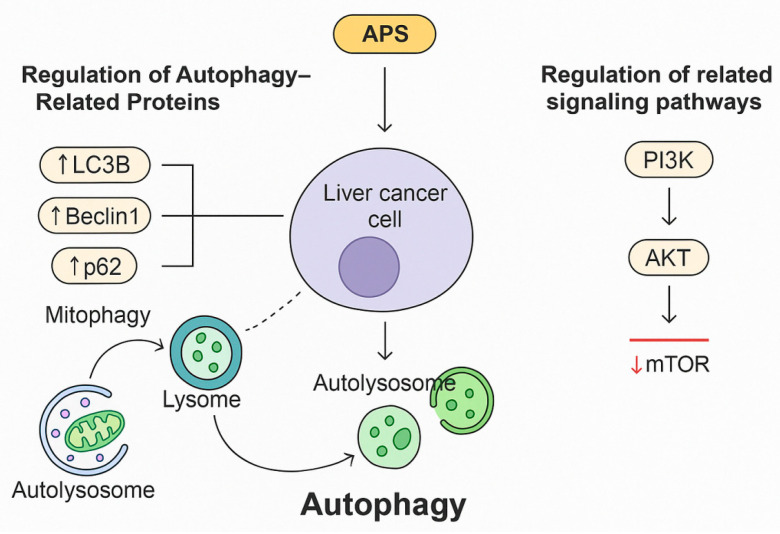
*Astragalus* polysaccharides (APS) regulate autophagy and inhibit liver cancer cell proliferation through the PI3K/AKT/mTOR signalling pathway.

Zhang et al. found that APS exert a significant inhibitory effect on liver cancer SMMC-7721 cells. APS reduced colony formation and cell viability in a concentration- and time-dependent manner and induced G0/G1 cell cycle arrest. Mechanistic studies showed that APS at a concentration of 3.00 mg/mL significantly upregulated the expression of autophagy-related proteins, such as LC3B, Beclin1, and p62, and activated mitophagy. Furthermore, when autophagy inhibitors (3-MA and Baf) were applied, the antitumour effects of APS were abolished, confirming that its inhibitory activity is mediated by autophagy [55]. This provides experimental evidence for the potential application of APS in liver cancer therapy and confirms autophagy as a key molecular target (Figure 4). Figure 4 illustrates the dual regulatory role of APS on autophagy and apoptosis in liver cancer cells. APS have been shown to upregulate autophagy-related proteins, including LC3B, Beclin-1, and p62, which contribute to mitophagy activation. APS treatment leads to G0/G1 phase arrest and reduced cell viability in SMMC-7721 cells. Moreover, when autophagy inhibitors such as 3-MA or bafilomycin (Baf) are introduced, the antitumour effect of APS is abolished, suggesting that autophagy is a key mechanism underlying APS-mediated cytotoxicity. Furthermore, APS inhibit the PI3K/AKT/mTOR signalling pathway, which is crucial for cell proliferation and survival, thereby enhancing autophagic flux and promoting mitochondria-dependent apoptosis. These findings support the potential of APS as therapeutic agents targeting autophagy and PI3K/AKT/mTOR pathways in hepatocellular carcinoma.

#### 3.3.2. Regulation of Related Signalling Pathways

The PI3K/AKT signalling pathway, primarily relayed through mTOR, plays a crucial role in regulating cell proliferation. It interacts with various proteins to form different functional complexes that precisely regulate fundamental biological processes within the cell [56]. One study investigated the inhibitory effects and underlying mechanisms of APS (0, 25, 50, and 100 μg/mL) on the proliferation of human liver cancer HepG2 cells over 24 h, with or without the Akt inhibitor LY294002 (10 μmol/L). MTT assays, Western blot analysis, and flow cytometry demonstrated that APS inhibited cell viability in a dose-dependent manner, induced G1 phase arrest, and triggered apoptosis (with a maximum apoptosis rate of 41.92%). APS also promoted autophagy by upregulating LC3B and downregulating LC3A and p62, while concurrently inhibiting the Akt/p-Akt signalling pathway. The combination of APS with LY294002 further enhanced apoptosis (apoptosis rate of 58.16%), indicating that APS suppresses hepatoma cell proliferation via the dual actions of autophagy activation and Akt pathway inhibition, thereby offering a potential therapeutic target for liver cancer [57].

### 3.4. Inhibition of the Epithelial–Mesenchymal Transition (EMT) and Metastasis

APS suppress the EMT and the metastatic potential in liver cancer models by modulating EMT-related markers and the key signalling pathways. The EMT is a fundamental biological process by which epithelial cells acquire mesenchymal, fibroblast-like properties, enabling enhanced migratory capacity, invasiveness, and resistance to apoptosis. This process plays a pivotal role in tumour progression and metastasis. APS have been shown to inhibit the EMT in hepatocellular carcinoma cells by upregulating epithelial markers such as E-cadherin and concurrently downregulating mesenchymal markers, including N-cadherin and vimentin. These molecular changes result in reduced invasiveness and impaired migratory behaviour of liver cancer cells [58]. In this study, the authors investigated the effect of APS, alone and in combination with 5-fluorouracil, on the EMT of hepatocellular carcinoma HepG2 cells. Using an MTT assay, a Transwell migration assay, RT-qPCR, and Western blotting, the researchers found that both APS alone and the APS + 5-FU combination inhibited the proliferation and migration of HepG2 cells. These treatments upregulated the expression of E-cadherin while downregulating the expression of vimentin and chemokine receptor 4, with the combination therapy showing superior efficacy compared to either agent alone. The most effective concentration was 785.26 μmol/L APS combined with 23.90 μmol/L 5-FU, with a treatment duration of 24 h. These results suggest that the combination may exert a synergistic inhibitory effect on hepatoma cell growth and metastasis by suppressing the EMT.

Mechanistically, the anti-EMT effects of APS are associated with the modulation of several signalling cascades known to regulate tumour progression. Notably, APS downregulate the activity of the Janus kinase/signal transducer and activator of transcription (JAK/STAT) pathway [59] and suppress Wnt/β-catenin signalling [51,52], both of which are implicated in EMT induction and cancer metastasis. In some models, inhibition of the TGF-β/Smad signalling pathway by APS has also been observed, further contributing to the reversal of EMT phenotypes. Through its regulatory influence on these pathways and associated biomarkers, APS effectively hinder the EMT, thereby attenuating the metastatic capacity of liver cancer cells and reinforcing their therapeutic promise in hepatocellular carcinoma management.

Xu et al. demonstrated that APS (200–400 mg/L) significantly inhibited the invasion (by 19–47%) and metastasis (by 30–43%) of liver cancer SMMC-7721 cells, primarily by downregulating the JAK/STAT signalling pathway, as indicated by reduced p-STAT3/STAT3 and p-STAT5/STAT5 ratios. The most pronounced effect was observed at a concentration of 300 mg/L. In this study, the authors assessed the antimetastatic effects of APS using scratch assays, Transwell invasion assays, and Western blot analysis. APS treatment inhibited cell migration and invasion in a concentration-dependent manner. The scratch healing rate declined from 91.35% ± 6.14% at 48 h to 52.12% ± 4.62%, and the number of invading cells decreased from 143 ± 15 to 76 ± 11 at 24 h. Mechanistically, APS suppressed the phosphorylation of STAT3 and STAT5. Moreover, co-treatment with the JAK inhibitor AG490 further enhanced this inhibitory effect, while the JAK/STAT activator colivelin reversed it. These findings confirm that APS inhibit the invasion and metastasis of hepatoma cells by targeting the JAK/STAT signalling pathway [59].

**Figure 5:**
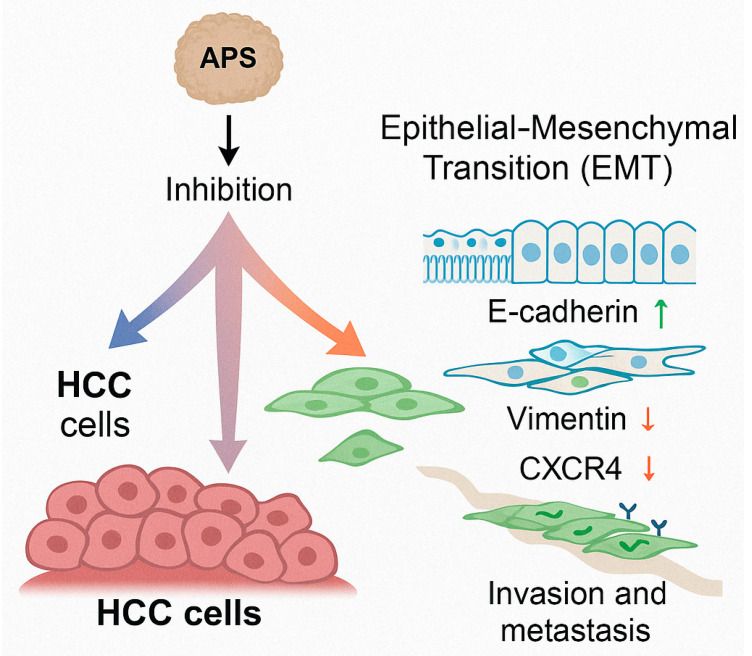
Inhibition of liver cancer cell invasion and metastasis by *Astragalus* polysaccharides (APS).

Hepatocellular carcinoma is characterised by its high invasiveness and metastatic potential, which significantly contribute to the poor prognosis observed in clinical oncology [60]. Studies have revealed that the EMT is a critical mechanism by which tumour cells acquire migratory and invasive capabilities [61]. Bai et al. [58] investigated the effect of APS on the EMT in hepatocellular carcinoma HepG2 cells and demonstrated that APS inhibited cell proliferation and invasion in a dose-dependent manner. Mechanistically, APS upregulated the epithelial marker E-cadherin while downregulating the mesenchymal marker vimentin and the chemokine receptor CXCR4 at both mRNA and protein levels, suggesting that APS suppress liver cancer cell growth and metastasis by inhibiting the EMT process (Figure 5), which illustrates the inhibitory role of APS on the EMT and metastatic progression in hepatocellular carcinoma (HCC) cells. APS treatment suppresses the EMT process by upregulating epithelial markers such as E-cadherin while downregulating mesenchymal markers, including vimentin and chemokine receptor CXCR4. This regulation of EMT-related molecules contributes to the inhibition of tumour cell invasion and metastasis. Thereby, APS limit the migratory and invasive behaviour of HCC cells, underscoring its therapeutic potential in preventing liver cancer dissemination.

### 3.5. Modulation of the Immune Response

APS exert robust immunomodulatory effects that contribute significantly to their anti-liver cancer activity. These effects span both the innate and adaptive arms of the immune system, thereby enhancing the host’s antitumour immunity. APS have been shown to stimulate innate immune responses by promoting macrophage activation and enhancing the phagocytic capacity of mononuclear cells. Furthermore, APS facilitate the maturation of dendritic cells, which are essential antigen-presenting cells, leading to increased secretion of pro-inflammatory cytokines such as interleukin-2 (IL-2), tumour necrosis factor alpha (TNF-α), and interferon gamma (IFN-γ) [32,33]. These cytokines play key roles in orchestrating antitumour responses and activating effector immune cells. In adaptive immunity, APS enhance the function of cytotoxic CD8^+^ T lymphocytes and reduce the population of immunosuppressive regulatory T cells (Tregs), thereby reversing tumour-induced immune tolerance [36]. Moreover, APS interfere with immune checkpoint signalling by downregulating Programmed death-ligand 1 (PD-L1) expression on tumour cells. Mechanistically, this effect is mediated via the miR-133a-3p/MSN axis, which destabilises PD-L1 and restores T cell-mediated cytotoxicity against liver cancer cells [62]. In this study, the anti-liver cancer mechanism of APS was investigated using a SMMC-7721 tumour-bearing BALB/c mouse model and hepatoma cell lines (SMMC-7721 and Huh-7). The results showed that APS, administered by intraperitoneal injection at doses of 100–400 mg/kg for 12 days or by pre-treating cells at concentrations of 0.1–1 mg/mL for 4 h, inhibited tumour growth in a dose-dependent manner, downregulated PD-L1 expression, and increased CD8^+^ T cell infiltration. Mechanistically, APS reduced the stability of PD-L1 and reversed IFN-γ-induced immunosuppression by upregulating miR-133a-3p, which targets the 3′UTR of MSN, thereby inhibiting both MSN protein expression and its phosphorylated form (p-MSN). The regulatory role of the miR-133a-3p/MSN/PD-L1 signalling axis was confirmed through flow cytometry, Western blotting, qRT-PCR, and dual-luciferase reporter assays, providing a novel target for liver cancer immunotherapy.

Additionally, APS demonstrate potential to enhance chimeric antigen receptor T cell (CAR-T) immunotherapy. Zhang et al. [63] reported that APS support CAR-T cell function by promoting the formation and maintenance of CD122^+^/CXCR3^+^/PD-1 memory T cell subsets, reducing the frequency of PD-1^+^ exhausted T cells, and increasing the expression of chemokines, such as CXCL9 and CXCL10, within the tumour microenvironment. Autophagy-related protein regulation was studied using two HCC mouse models. In the subcutaneous model, NOD/SCID mice received Huh7 or HepG2 cells, and treatments began five days later. APS (50 mg/kg/day) were administered orally, and CAR-T cells (5 × 10^6^) were injected on day 10 after cyclophosphamide (200 mg/kg) pre-treatment. In the orthotopic model using Huh7-luc cells, APS were started one week post-implantation, followed by CAR-T therapy in the second week. The combined APS and CAR-T treatment significantly inhibited tumour growth compared to monotherapies. Flow cytometry showed increased total and CD8^+^ CAR-T cell infiltration in the APS+CAR-T group, suggesting enhanced CAR-T cell persistence. These results indicate that APS potentiate CAR-T efficacy and may modulate autophagy-related protein expression in tumour-bearing mice. These effects culminate in improved proliferation, tumour infiltration, and sustained activity of CAR-T cells in hepatocellular carcinoma models. Collectively, these findings suggest that APS serve as potent immune enhancers in liver cancer therapy, capable of reshaping the tumour immune microenvironment and potentiating both natural and engineered antitumour immune responses.

#### 3.5.1. Enhancement of Immune Organ Indices

The thymus and the spleen are central organs of the immune system, and their status directly reflects the immune function [64]. Lai et al. [32] investigated the antitumour effects of *Astragalus* polysaccharides (APS) in H22 tumour-bearing mice and found that APS at doses of 100, 200, and 400 mg/kg significantly inhibited tumour growth, with the highest inhibition rate of 59.01% observed in the 400 mg/kg group. In this study, liver cancer cells were inoculated into mice, and treatment with APS commenced 24 h post-inoculation. APS were administered once daily by gavage at the specified doses: 100 mg/kg (low-dose group), 200 mg/kg (medium-dose group), and 400 mg/kg (high-dose group), continuing until day 15. On day 16, the mice were sacrificed for analysis. The results showed that APS not only suppressed tumour growth, but also significantly increased the thymus and spleen indices and enhanced the production of serum cytokines IL-2, IL-6, and TNF-α. These findings suggest that APS exert their antitumour effects, at least in part, through modulation of the immune response.

Similarly, Yang et al. [33] reported that APS at doses of 100 and 400 mg/kg significantly suppressed tumour growth, increased body weight and immune organ indices in tumour-bearing mice, enhanced macrophage phagocytic function, and promoted the secretion of IL-2, IL-12, and TNF-α, while reducing IL-10 levels. These findings highlight the potential of APS in boosting host immune responses and support its development as a safe anticancer agent. In this study, female BALB/c mice were used. Twenty-four hours after inoculation with H22 hepatoma cells, the mice were randomly assigned to four groups: a model group (treated with normal saline), a 5-FU group (20 mg/kg), a low-dose APS group (100 mg/kg), and a high-dose APS group (400 mg/kg). All treatments were administered via daily gavage for 10 days. Assessment indicators included tumour weight and inhibition rate, spleen and thymus indices, serum cytokine levels (IL-2, IL-12, TNF-α, IL-10), as well as the phagocytic rate and phagocytic index of peritoneal macrophages. Statistical analysis was performed using one-way ANOVA and *t*-tests. The results confirmed that APS inhibited tumour growth by modulating the immune function, as evidenced by the increased thymus and spleen indices and elevated levels of IL-2, IL-12, and TNF-α. These outcomes reinforce the immunomodulatory and antitumour potential of APS.

#### 3.5.2. Inhibition of Immune Checkpoints

PD-L1 is a protein expressed on tumour and immune cells that binds to the PD-1 receptors on T cells, suppressing their activity and allowing tumour cells to evade immune surveillance [65]. MicroRNAs (miRNAs) are endogenous, non-coding single-stranded RNAs with key roles in tumour progression. Some miRNAs act as tumour suppressors, while others function as oncogenes [66]. Moesin (MSN), a protein involved in cytoskeletal rearrangement, has been found to correlate with tumour progression in various cancers [67]. He et al. [62] demonstrated that APS (100–400 mg/kg) could inhibit liver cancer growth by reducing PD-L1 expression and PD-L1-mediated immunosuppression, with IC_50_ equal to 4.2 mg/mL. Mechanistically, APS upregulate miR-133a-3p, which suppresses its target gene MSN, thereby destabilizing PD-L1 and enhancing immune-mediated tumour cell killing. This highlights a novel pathway for APS in liver cancer therapy.

#### 3.5.3. Optimisation of CAR-T Cell Therapy for Liver Cancer

CAR-T therapy is an advanced immunotherapy that involves genetically modifying a patient’s T cells to recognise and destroy tumour cells [68]. Zhang et al. found that APS can enhance the efficacy of CAR-T therapy in liver cancer by activating the STAT5 signalling pathway, promoting the formation and maintenance of CD122^+^/CXCR3^+^/PD-1 memory T cells, reducing inhibitory PD-1^+^ subpopulations, and increasing the expression of CXCL9/CXCL10 chemokines in the tumour microenvironment. This facilitates CAR-T cell proliferation, migration, and tumour infiltration. Both in vivo and in vitro experiments showed that APS combined with CAR-T significantly inhibited tumour growth in Huh7 and HepG2 models and improved the persistence and functionality of CD8^+^ CAR-T cells, offering a new strategy for liver cancer immunotherapy with the appropriate concentration of 800 μg/mL [63].

#### 3.5.4. Regulation of Macrophage Polarisation

Macrophages play a critical role in immune defence and tissue homeostasis [69]. In tumours, macrophages exhibit functional plasticity and can polarise into pro-inflammatory M1 or protumour M2 phenotypes. M1 macrophages produce reactive oxygen species and cytokines that kill tumour cells and activate other immune responses [70], whereas M2 macrophages promote tumour growth, angiogenesis, and immune evasion [71]. Li et al. [72] demonstrated that APS inhibit M2 polarisation of TAMs. In vitro treatment of TAMs with 16 mg/mL APS increased the expression of M1 markers (iNOS, IL-1β, TNF-α) and reduced M2 markers (IL-10, Arg-1). Co-cultured MHCC97H and Huh7 cells showed suppressed proliferation, migration, and invasion. In vivo, APS (50–200 mg/kg) significantly reduced tumour volume and weight in tumour-bearing mice, with increased M1 and decreased M2 macrophages in tumour tissues. This suggests that APS can remodel the tumour microenvironment via TAM reprogramming to inhibit liver cancer progression.

#### 3.5.5. Regulation of Regulatory T Cells (Tregs)

Tregs, such as CD4^+^CD25^+^ Tregs, are immunosuppressive T cell subsets responsible for maintaining immune tolerance, primarily through the secretion of IL-10 and TGF-β, or by directly inhibiting the function of effector T cells. These effector immune cells are suppressed via multiple mechanisms regulated by the transcription factor Foxp3—mechanisms that are frequently co-opted by tumours to escape immune surveillance. Within the tumour microenvironment, Tregs play a crucial role in facilitating immune evasion and are strongly associated with tumour progression and poor clinical prognosis [73,74]. APS have been shown to restore cytokine balance within the tumour microenvironment and downregulate FOXP3 mRNA expression, thereby attenuating the immunosuppressive activity of CD4^+^CD25^+^ Tregs. Additionally, APS may interfere with the CXCR4/CXCL12 (SDF-1) chemokine axis, reducing Treg infiltration into tumour sites and consequently enhancing antitumour immune responses. These effects collectively contribute to delayed liver cancer progression and improved survival outcomes [36]. In this study, surgical tissue and peripheral blood samples were collected from 31 patients with liver cancer. CD4^+^CD25^+^ regulatory T (Treg) cells were isolated using flow cytometry, and various concentrations of *Astragalus* polysaccharides (APS; 10–200 μg/mL) were applied for in vitro treatment over 24, 48, and 72 h. Through a combination of MTT assays, ELISA, qRT-PCR, and Transwell migration assays, the results demonstrated that APS inhibited Treg cell proliferation in a dose- and time-dependent manner. Additionally, APS upregulated the pro-inflammatory cytokine IFN-γ, downregulated the anti-inflammatory cytokines IL-10 and IL-4, and decreased the expression of FOXP3 mRNA. Furthermore, APS inhibited the migration of Treg cells to the tumour microenvironment by blocking the SDF-1/CXCR4 signalling pathway, thereby reversing the immunosuppressive microenvironment in liver cancer and offering a novel strategy for immunotherapy.

**Figure 6:**
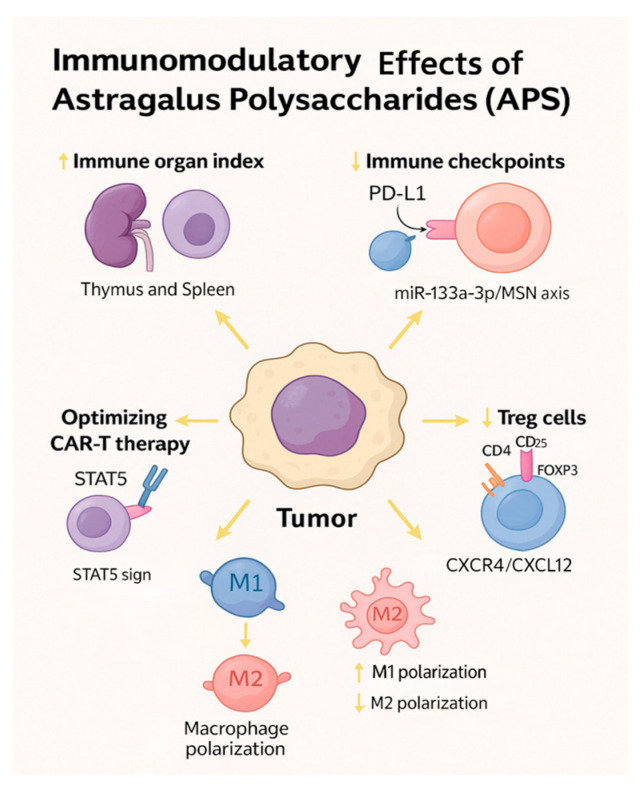
Immunomodulatory mechanisms of *Astragalus* polysaccharides (APS) in liver cancer.

Figure 6 illustrates the multifaceted immunomodulatory effects of APS against liver cancer. APS enhance the immune organ function by increasing the spleen and thymus indices and promoting the secretion of immune cytokines such as interleukin (IL)-2, IL-6, IL-12, and tumour necrosis factor alpha (TNF-α), while reducing immunosuppressive cytokines such as IL-10. APS inhibit immune checkpoints by downregulating PD-L1 via the miR-133a-3p/MSN axis, thereby restoring T cell-mediated cytotoxicity. They also enhance the efficacy of chimeric antigen receptor T cell (CAR-T) therapy by activating the STAT5 signalling pathway, promoting the formation of CD122^+^/CXCR3^+^/PD-1 memory T cells, and increasing tumour infiltration through elevated CXCL9/CXCL10 expression. Additionally, APS promote macrophage M1 polarisation while inhibiting M2 polarisation, as indicated by the upregulation of iNOS, IL-1β, TNF-α, and downregulation of IL-10 and Arg-1, respectively. APS also reduce the proportion and suppressive function of Tregs by inhibiting FOXP3 expression and blocking CXCR4/CXCL12-mediated chemotaxis. Collectively, these mechanisms contribute to reversing immune suppression and reshaping the tumour immune microenvironment in hepatocellular carcinoma.

### 3.6. Regulation of the Tumour Microenvironment

The tumour microenvironment plays a pivotal role in the initiation, progression, and therapeutic resistance of hepatocellular carcinoma. APS have been increasingly recognised for their ability to remodel the tumour microenvironment in favour of tumour suppression. One of the critical mechanisms through which APS influence the tumour microenvironment is the modulation of TAMs [72,75,76]. APS promote the polarisation of TAMs from the M2 phenotype—which supports tumour growth, angiogenesis, and immune suppression—to the M1 phenotype, which possesses pro-inflammatory and antitumour properties [72]. In this study, THP-1 monocytes were induced into TAMs and treated with APS at 0, 8, and 16 mg/mL for 24 h. APS at 16 mg/mL significantly enhanced M1 markers (iNOS, TNF-α) and reduced M2 markers (IL-10, Arg-1). When co-cultured with MHCC97H or Huh7 cells, APS inhibited their proliferation, migration, and invasion. In vivo, Huh7 tumour-bearing BALB/c nude mice (4–6 weeks old, 15–20 g) were treated with intraperitoneal injections of APS (50–200 mg/kg) for 30 days. APS reduced tumour volume dose-dependently, with a 50% reduction at 200 mg/kg, increased M1 macrophages (CD86^+^), and decreased M2 macrophages (CD206^+^), indicating that APS reshape the tumour immune microenvironment by inhibiting M2 polarisation.

**Figure 7:**
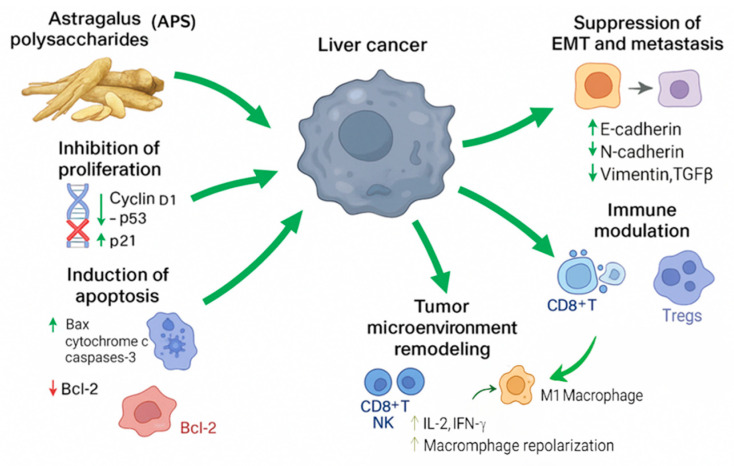
Multifaceted antitumour mechanisms of *Astragalus* polysaccharides (APS) in liver cancer. This figure illustrates the diverse mechanisms through which APS exert antitumour effects in hepatocellular carcinoma (HCC). APS inhibit tumour proliferation by downregulating cyclin D1 and p53 and upregulating p21, leading to cell cycle arrest. They promote apoptosis via mitochondrial pathways, characterised by increased Bax, cytochrome c, and caspase-3 levels and reduced Bcl-2 expression. APS also suppress the epithelial–mesenchymal transition (EMT) and metastasis by upregulating E-cadherin and downregulating mesenchymal markers such as N-cadherin, vimentin, and TGF-β. Immunomodulatory effects include activation of CD8^+^ T cells and natural killer (NK) cells, increased secretion of IL-2 and IFN-γ, and reduced Treg activity. Furthermore, APS remodel the tumour microenvironment (TME) by repolarising TAMs from the protumour M2 phenotype to the antitumour M1 phenotype and downregulating angiogenic factors such as VEGF and HIF-1α. Together, these mechanisms position APS as promising multifunctional agents for integrative liver cancer therapy.

**Figure 8:**
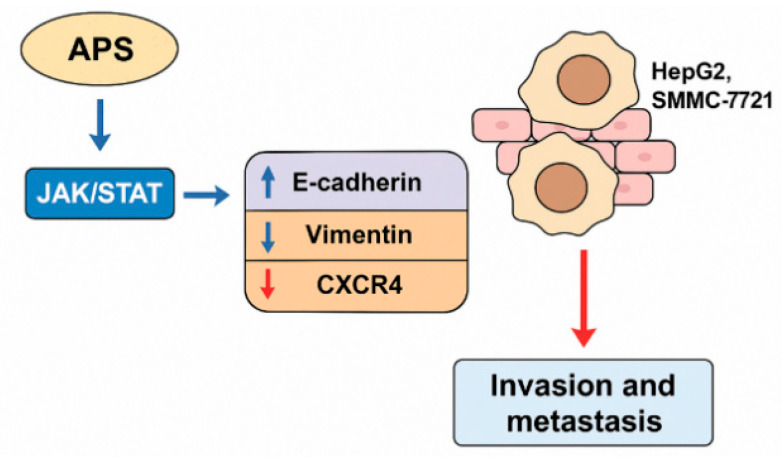
Inhibition of liver cancer cell invasion and metastasis by *Astragalus* polysaccharides (APS). This figure illustrates the inhibitory effects of APS on the invasion and metastasis of liver cancer cells, specifically HepG2 and SMMC-7721 cell lines. APS modulate the JAK/STAT signalling pathway, leading to upregulation of the epithelial marker E-cadherin and downregulation of the mesenchymal markers vimentin and CXCR4. These changes inhibit the EMT, suppress cell migration and invasion, and reduce the metastatic potential. The data support the potential of APS as natural agents targeting metastasis in hepatocellular carcinoma.

This phenotypic shift contributes to an enhanced immune response against liver cancer cells. Moreover, APS exert anti-angiogenic effects by downregulating vascular endothelial growth factor and other angiogenesis-promoting factors, thereby reducing tumour vascularisation and nutrient supply. APS have also been shown to alleviate tumour hypoxia and inhibit the deposition of extracellular matrix components that facilitate stroma–tumour interactions and metastatic dissemination. In combination, these actions suggest that APS not only target tumour cells directly, but also modify the supportive environment in which they thrive, creating a more hostile milieu for tumour survival and progression. This multifaceted regulation of the tumour microenvironment underscores the therapeutic potential of APS as part of an integrative strategy for hepatocellular carcinoma management. Table 2 and Figure 7 and Figure 8 summarise the main antitumor mechanisms of APS in liver cancer. APS exert multiple biological effects, including inhibition of proliferation, induction of apoptosis, modulation of autophagy, suppression of metastasis, immune regulation, and remodelling of the tumour microenvironment.

## 4. Mechanisms of Synergistic Therapies: APS Enhance Standard Chemotherapeutics and Reverse Resistance

Novel delivery strategies, including APS-loaded selenium nanoparticles and liposomes, further improve bioavailability and tumour targeting. Combined treatments result in enhanced apoptosis, reduced adverse effects, and improved patient outcomes. The combined application of APS with chemotherapeutic agents has increasingly demonstrated its potential synergistic effects in liver cancer treatment. Studies have shown that APS can enhance the efficacy of chemotherapy while reducing the associated toxicity, thereby offering more effective treatment strategies for liver cancer patients [77].

### 4.1. Antitumour Applications of APS-Modified Selenium Nanoparticle Composites

Nanotechnology has been widely applied in the biomedical field, and selenium nanoparticles (SeNPs) have emerged as a focal point for research in targeted drug delivery for cancer treatment due to their unique anticancer mechanisms, favourable pharmaceutical properties, and inhibitory effects on tumour cells [78]. The preparation of polysaccharide–SeNP composites typically involves using sodium selenite (Na_2_SeO_3_) as the precursor and ascorbic acid (Vc) as the reducing agent, with synthesis carried out via a chemical reduction method in a polysaccharide solution. Two primary methods are employed: either pre-mixing the polysaccharide with sodium selenite or pre-mixing the polysaccharide with the reducing agent, followed by the addition of sodium selenite [79]. Ji et al. [80] provided important insights into the application of nanotechnology in liver cancer treatment by developing a novel composite of alcohol-soluble polysaccharides extracted from *Astragalus membranaceus* (AASP) modified with selenium nanoparticles (SeNPs), termed AASP–SeNPs. This composite was synthesised through the reaction of sodium selenite with AASP at a mass ratio of 1:20, resulting in uniformly spherical nanoparticles with an average diameter of 49.80 nm. These nanoparticles exhibited excellent dispersibility and stability in aqueous solutions.

**Figure 9:**
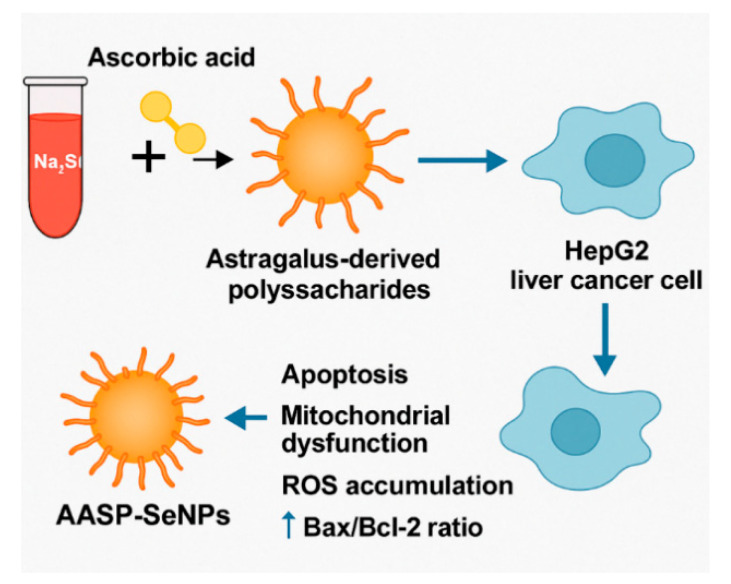
Schematic illustration of the synthesis and mechanism of action of *Astragalus* polysaccharide-modified selenium nanoparticles (AASP–SeNPs) in liver cancer therapy. This figure shows that AASP–SeNPs are synthesised by reducing sodium selenite (Na_2_SeO_3_) with ascorbic acid in an *Astragalus* polysaccharide solution, producing spherical, monodisperse nanoparticles of approximately 50 nm in diameter. After being internalised by human liver cancer (HepG2) cells, AASP–SeNPs induce the accumulation of reactive oxygen species and a loss of the mitochondrial membrane potential (ΔΨm). This loss of ΔΨm triggers the release of cytochrome c from mitochondria. These events are accompanied by an increase in the proapoptotic protein Bax and a decrease in the antiapoptotic protein Bcl-2, shifting the balance toward apoptosis.

The AASP–SeNPs displayed a significant dose-dependent inhibitory effect on HepG2 cells, with the inhibition rate increasing markedly as the concentration rose from 25 to 800 μg/mL and the treatment period extended from 24 to 48 h. At a concentration of 400 μg/mL, the induced apoptosis rate reached as high as 55.43%. Mechanistic investigations revealed that AASP–SeNPs elevated intracellular ROS levels and reduced the mitochondrial membrane potential (∆Ψm). This led to mitochondrial dysfunction and triggered the release of cytochrome c into the cytoplasm, accompanied by upregulation of the proapoptotic protein Bax and downregulation of the antiapoptotic protein Bcl-2. These changes collectively activated the caspase cascade, ultimately promoting apoptosis in HepG2 cells. The study underscores the potential of AASP–SeNPs as a promising therapeutic strategy for liver cancer by integrating the bioactivity of traditional Chinese medicine polysaccharides with the targeted delivery advantages of nanotechnology (Figure 9).

### 4.2. Combination with Doxorubicin

Doxorubicin, an anthracycline antibiotic, is one of the commonly used chemotherapeutic agents in the treatment of liver cancer [81]. Studies have revealed that APS enhance doxorubicin-induced endoplasmic reticulum (ER) stress by reducing O-GlcNAcylation levels, thereby promoting apoptosis of liver cancer cells. In vitro experiments demonstrated that APS enhanced the inhibitory effect of doxorubicin on Hep3B cells in a dose-dependent manner, significantly increased apoptosis, downregulated O-GlcNAc transferase (OGT), and upregulated O-GlcNAcase (OGA) expression. In vivo tumour-bearing mouse models showed that combination therapy significantly enhanced ER stress (indicated by the PERK/eIF2α/CHOP pathway activation) and the expression of proapoptotic proteins such as cleaved caspase-3, Bax. In Li’s in vitro experiment, the combination of APS at concentrations of 2, 10, and 50 mg/L with 1 μM doxorubicin resulted in a dose-dependent decrease in cell viability. For example, the 10 mg/L APS + doxorubicin group showed a reduction in cell viability to 60%, which was further decreased in the 50 mg/L group. At 50 mg/L, APS were subsequently used to investigate O-GlcNAcylation modification and endoplasmic reticulum (ER) stress-related pathways. The results demonstrated that this concentration significantly downregulated the expression of O-linked N-acetylglucosamine transferase (OGT) and upregulated the expression of O-GlcNAcase (OGA) [37].

Tian et al. [38] investigated the effects of APS on doxorubicin-resistant H22 liver cancer cells, focusing on the P-glycoprotein (P-GP) efflux function and expression. While APS alone exhibited limited antitumour activity (IC_50_ = 251.77 mg/L after 24–72 h), pre-treatment with APS (0.8–500 mg/L for 24 h) significantly enhanced the cytotoxicity of chemotherapeutic agents such as doxorubicin and cisplatin in a concentration- and time-dependent manner. For example, the IC_50_ of doxorubicin was reduced from 6.16 μg/mL to 2.61 μg/mL with 500 mg/L APS and further decreased to 1.55 μg/mL after 72 h of combined treatment. Mechanistically, APS reduced the MDR1 mRNA and P-GP protein expression, inhibited P-GP efflux activity, and increased the intracellular accumulation of rhodamine-123, with fluorescence intensity in the 500 mg/L group being six times higher than in the control. This effect strengthened with prolonged exposure. These findings indicate that APS can reverse multidrug resistance by inhibiting the P-GP function and expression, thereby enhancing the sensitivity of resistant tumour cells to chemotherapy and enabling effective tumour inhibition at lower doxorubicin concentrations.

Liposomal drug delivery, which leverages a lipid bilayer structure, offers targeted transport, sustained release, and reduced toxicity of chemotherapeutics [82]. In liver cancer treatment, it shows notable advantages. Jiang et al. [83] developed liver-targeted liposomes co-loaded with APS and doxorubicin using ethanol injection (particle size of 168.9 nm, good dispersion), demonstrating effective uptake by HepG2 cells, nuclear accumulation, and superior antiproliferative activity compared to single-agent liposomes—offering a new strategy for TCM–chemotherapy co-delivery in liver cancer.

### 4.3. Combination with Apatinib

Apatinib is a novel small-molecule antiangiogenic drug that inhibits liver cancer progression by regulating apoptosis, controlling cell viability, and suppressing migration [84,85]. One study showed that APS, apatinib, and their combination reduce the survival, migration, and invasion of Hep3B liver cancer cells, increase apoptosis, and lower tumour marker levels of CA199 and CA724 [85]. In that study, the effects of APS combined with apatinib on the Hep3B hepatoma cell line were investigated using CCK-8 assays, flow cytometry, Transwell migration and invasion assays, and ELISA. The combined treatment with 200 mg/L APS and 20 μmol/L apatinib significantly inhibited cell proliferation (survival rate: 64.22%), induced apoptosis (37.73%), and reduced the number of migratory and invasive cells to 114 and 82, respectively. It also decreased the CA199 and CA724 levels to 5.42 U/mL and 26.16 U/mL. These effects were significantly greater than those observed with either drug alone, indicating that APS synergistically enhance the antitumour effect of apatinib by inhibiting the proliferation, migration, and invasion of liver cancer cells, while also reducing tumour biomarker expression. This supports the potential of APS and apatinib as a promising combination for clinical therapy.

### 4.4. Combination with Cisplatin

Cisplatin is widely used in systemic therapy for liver cancer, particularly in advanced stages [86]. Zhao et al. [87] found that APS inhibited BEL-7404 human liver cancer cell growth in a concentration-dependent manner and showed stronger cytotoxicity when combined with cisplatin. The combination of APS with cisplatin significantly changed the IC_50_ values of both agents: APS—from 1000 μg/mL to 690 μg/mL, cisplatin—a 2.5-fold increase in efficacy at 7.5 μg/mL. This synergistic interaction markedly enhanced the cytotoxic efficiency compared to either monotherapy alone. The combination reduced the 24 h IC_50_ of cisplatin and increased the killing efficiency by 1.4 times. Flow cytometry showed cell cycle arrest in the G1 phase and a sub-G1 apoptotic peak, with the apoptosis rate reaching 54.76%, confirming the synergistic antitumour effect of APS and cisplatin.

### 4.5. Combination with Transarterial Chemoembolisation (TACE)

TACE is a crucial treatment for intermediate to advanced-stage hepatocellular carcinoma. It delivers localised, high-concentration chemotherapy by embolizing the tumour’s arterial supply [39,88]. Li et al. [39] found that APS (250 mg), when combined with TACE (oxaliplatin, 200 mg; fluorouracil glycoside, 500–1000 mg; doxorubicin, 30–60 mg) and targeted therapy, significantly reduced levels of alpha-fetoprotein and total bilirubin, suggesting hepatoprotective effects. APS also alleviated adverse treatment reactions and improved patients’ quality of life, offering a novel clinical approach for advanced HCC. In this study, the efficacy of APS combined with TACE was evaluated through both clinical and mechanistic investigations. Among 132 patients, the APS-treated group showed prolonged overall survival (OS) of 17 months compared to 12 months in the control group, and progression-free survival (PFS) was extended to 10 months versus 8.5 months in the control. Additionally, serum markers such as alpha-fetoprotein and total bilirubin were significantly improved, and adverse reactions were reduced. Mechanistically, APS was found to inhibit hepatoma cell proliferation, migration, and invasion by modulating TAMs. Specifically, it promoted TAM polarisation towards the antitumour M1 phenotype (increasing from 8.4% to 75.4%) and suppressed the pro-tumour M2 phenotype (decreasing from 56.7% to 1.19%). These findings provide a new immunomodulatory strategy for the treatment of HCC.

### 4.6. Combination with 5-Fluorouracil (5-FU)

5-FU is a widely used antimetabolite chemotherapeutic agent that interferes with DNA/RNA synthesis and inhibits cancer cell proliferation [89]. Studies have shown that APS and 5-FU, alone or in combination, suppress proliferation and invasion of HepG2 cells, with the combined effect being superior in a dose-dependent manner. Mechanistically, combination treatment upregulated epithelial marker E-cadherin and downregulated mesenchymal markers vimentin and chemokine receptor CXCR4 at both the mRNA and protein levels, indicating inhibition of the EMT [58]. Mei et al. [90] demonstrated that APS can reverse 5-FU resistance in liver cancer cells by downregulating the expression of resistance-related genes GST-π and MDR1. In this study, the inhibitory effects of APS on the proliferation of the 5-FU-resistant liver cancer cell line Bel-7402/5-FU were assessed using CCK-8 assays, flow cytometry, and molecular analyses. APS exhibited a significant antiproliferative effect, with an IC_50_ of 0.6 mg/mL after 72 h of treatment. When administered for 2 to 7 days, both the APS monotherapy group (0.6 mg/mL) and the combination group (APS 0.6 mg/mL + 5-FU 10 μg/mL) showed significantly lower proliferation rates compared to the control and 5-FU-only groups. The strongest inhibitory effect was observed in the combination group after 7 days of treatment. After 72 h, the combination treatment reduced the proportions of cells in the S phase and the G2/M phase to 26.7% and 8.1%, respectively, decreased the proliferation index to 0.34, and increased the apoptosis rate to 26.1%. Mechanistically, both APS alone and in combination with 5-FU (after 48 h of treatment) significantly downregulated the mRNA and protein expression levels of GST-π and MDR1, with the greatest reduction observed in the combination group. These results indicate that APS enhance the sensitivity of hepatoma cells to 5-FU by reversing drug resistance and that combination therapy exerts synergistic antiproliferative and proapoptotic effects.

### 4.7. Combination with Cantharidin (CTD)

CTD, derived from blister beetles (*Mylabris* spp.), is a traditional antitumour agent with over 2000 years of use. However, its hepatotoxicity limits clinical application [91,92,93,94]. Huang et al. [94] demonstrated that APS alleviate CTD-induced subacute liver injury in mice by modulating glycerophospholipid metabolism and primary bile acid biosynthesis. In this study, a CTD-induced liver injury model was established using six-week-old male Kunming mice, randomly divided into three groups: a control group, a CTD model group (1 mg/kg CTD via intragastric administration), and an APS protection group (100 mg/kg APS pre-treatment via intragastric administration for 4 h prior to 1 mg/kg CTD), with treatments continuing for 14 days. The CTD model group exhibited significant liver injury, characterised by weight loss, increased liver index, elevated serum ALT, AST, ALP, and LDH levels, elevated hepatic MDA content, reduced SOD activity, and histological evidence of hepatocellular swelling, necrosis, and inflammatory infiltration. In contrast, the APS protection group showed a marked improvement in these parameters, indicating a protective effect against CTD-induced liver damage. Metabolomics analysis further revealed that CTD-induced liver injury was primarily associated with disruptions in glycerophospholipid metabolism, ABC transporter function, and choline metabolism. APS treatment reversed these metabolic disturbances by regulating pathways related to primary bile acid biosynthesis, glycerophospholipid metabolism, and bile secretion. These findings support the clinical potential of APS in mitigating CTD hepatotoxicity and provide a mechanistic basis for their hepatoprotective effects.

### 4.8. Combination with Docetaxel (DTX), Cyclophosphamide (CTX), and Epirubicin (EPI)

**Figure 10:**
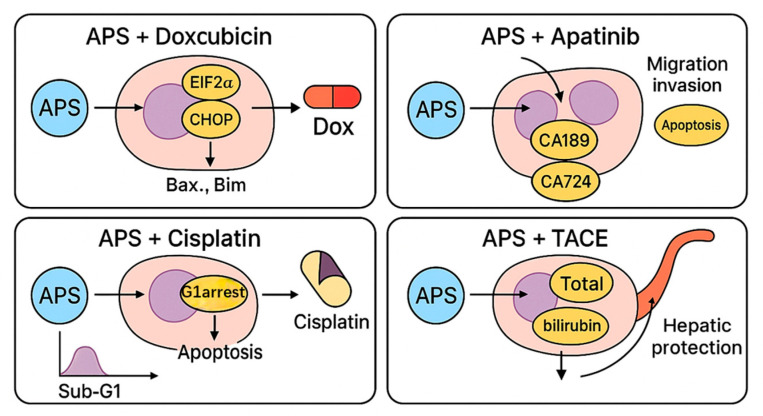
Synergistic effects of *Astragalus* polysaccharides (APS) with chemotherapeutic agents in liver cancer. This figure illustrates the synergistic actions of APS when used in combination with chemotherapeutic agents or interventional therapies in hepatocellular carcinoma (HCC). In the top left panel, APS enhance doxorubicin (Dox)-induced apoptosis through activation of endoplasmic reticulum (ER) stress pathways, including PERK, eIF2α, and CHOP, and increase expression of proapoptotic proteins Bax and Bim. The top right panel shows that APS combined with apatinib promote apoptosis and inhibit tumour migration and invasion, alongside reduced levels of tumour markers CA189 and CA724. In the bottom left panel, APS co-administered with cisplatin result in G1 cell cycle arrest, increased Sub-G1 cell population, and augmented apoptotic activity. The bottom right panel demonstrates that APS improve the therapeutic efficacy of transarterial chemoembolisation (TACE) by lowering serum alpha-fetoprotein (AFP) and total bilirubin levels, while also offering hepatic protection. Collectively, these data indicate that APS act as a multifunctional adjuvant capable of enhancing chemotherapeutic efficacy, reducing adverse effects, and modulating tumour-associated pathways in liver cancer treatment.

DTX, CTX, and EPI are commonly used chemotherapeutic agents for various malignancies. Despite their efficacy, they cause significant toxicity [95,96]. Liu et al. [97] investigated the protective effects of APS against liver injury induced by these chemotherapy drugs in mice. Male Kunming mice (7–8 weeks old) were divided into the control, chemotherapy-only, and chemotherapy-plus-APS groups. Liver injury models were established using CTX (10/20 mg/kg), DTX (0.5/2 mg/kg), and EPI (0.8/3 mg/kg), administered intraperitoneally every two days for 28 consecutive days. APS (100 mg/kg/day) were administered intraperitoneally during the last week (days 22–28) in the combination groups. The results showed that chemotherapy drugs induced dose-dependent liver damage. Mice in the high-dose groups, particularly with CTX, exhibited significant weight loss (weight gain of only 14.5%), marked increases in serum ALT and AST (ALT increased by 366.7%), and pathological changes, including hepatocyte swelling, necrosis, and mitochondrial injury. Among the three drugs, CTX caused the most severe hepatic toxicity. APS treatment significantly reduced serum ALT and AST levels (ALT decreased by 28.6% in the CTX+APS group), alleviated liver tissue damage, and downregulated the expression of caspase-3 by 28.3%, indicating reduced apoptosis. These findings suggest that APS protects against chemotherapy-induced liver injury, particularly that caused by CTX, through antiapoptotic mechanisms (Figure 10).

### 4.9. Antitumour Effects of the Compound Astragalus and Salvia Extract (CASE)

The compound *Astragalus* and *Salvia* extract (CASE) is composed of active constituents derived from *Astragalus membranaceus* and *Salvia miltiorrhiza*, including astragalosides, APS, and salvianolic acids. As traditional Chinese medicinal herbs, both *Astragalus* and *Salvia* exert therapeutic effects through multicomponent and multitarget mechanisms. Their extracts show broad prospects in disease treatment, particularly demonstrating significant potential in cancer therapy. Smads are a class of evolutionarily conserved signal transduction proteins and serve as core components of the transforming growth factor-β (TGF-β) superfamily signalling pathway. They play crucial roles in regulating cell growth, differentiation, apoptosis, and tumour development. Smad3 phosphorylation can be classified into C-terminal phosphorylation (pSmad3C), which is directly activated by TGF-β receptors and mediates tumour-suppressive signalling, and linker-region phosphorylation (pSmad3L), which is activated via the MAPK pathways (including ERK, JNK, and p38) and is associated with tumour progression [98,99,100].

Wu et al. [101] found that the CASE (80 μg/mL), which contains APS, could inhibit liver cancer cell migration and proliferation, promote apoptosis, and suppress tumour growth by promoting the conversion of pSmad3L to pSmad3C (a tumour-suppressing pathway) while inhibiting MAPK-dependent phosphorylation of pSmad3L (a tumour-promoting pathway). This regulation was associated with the upregulation of miR-145 and the downregulation of miR-21. Beyond its antitumour effects, the CASE also showed the ability to improve liver fibrosis and suppress liver cancer progression.

In a subsequent study, Boye et al. [102] demonstrated that the CASE inhibited the phosphorylation of MAPK subtypes, including ERK, JNK, and p38, in a dose- and time-dependent manner. This suppression of MAPK phosphorylation reduced the persistent activation of the MAPK pathway induced by DEN, thereby inhibiting MAPK-dependent linker-region phosphorylation of Smad2/3 (associated with carcinogenic non-canonical TGF-β signalling) and attenuating the nuclear translocation of Smad2/3. Simultaneously, the CASE suppressed the expression of Smad4 within the canonical TGF-β pathway and disrupted its nuclear transport mediated by importin-7 (Imp7), ultimately resulting in a significant downregulation of the downstream oncogene plasminogen activator inhibitor-1 (PAI-1).

**Figure 11:**
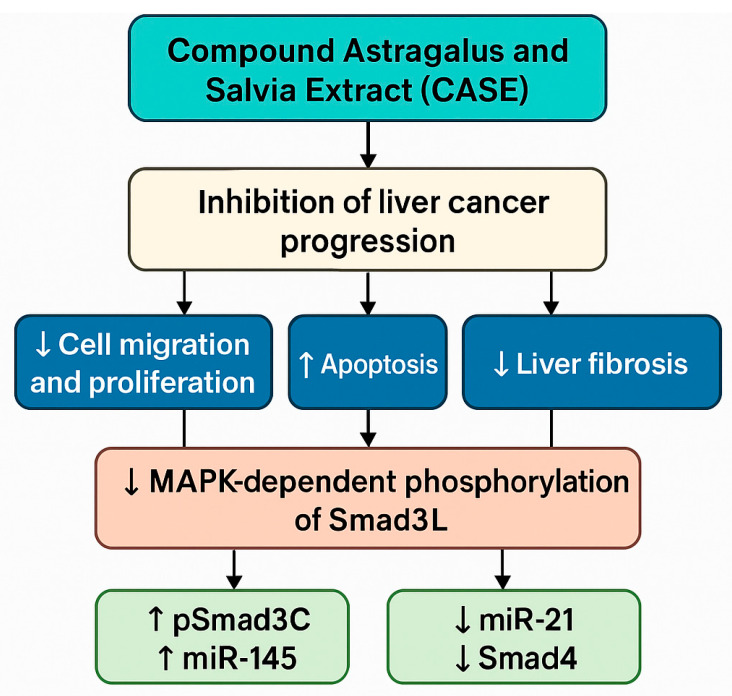
Mechanism of the compound *Astragalus* and *Salvia* extract (CASE) in inhibiting liver cancer progression via the MAPK–TGF-β/Smad pathway modulation.

These effects were consistently observed across multiple cell types, including HepG2 and hepatic stellate cells, and were validated in both in vitro and in vivo models (DEN-induced rat hepatocellular carcinoma). This study was the first to demonstrate that a natural compound could concurrently intervene in both canonical and non-canonical TGF-β pathways and achieve multitarget inhibition of hepatocellular carcinoma via comprehensive anti-MAPK activity [102]. Importantly, this work provides molecular evidence supporting the potential of targeting liver cancer progression through MAPK–TGF-β/Smad crosstalk using the CASE, offering mechanistic insights that advance the modernisation of TCM (Figure 11).

## 5. Conclusions and Future Perspectives

APS, the key bioactive constituents of *Astragalus membranaceus*, have demonstrated considerable promise as multifunctional agents in the prevention and treatment of hepatocellular carcinoma. As summarised in this review, APS exert potent antitumour activities through diverse mechanisms, including the induction of apoptosis, inhibition of cell proliferation, suppression of the EMT, modulation of autophagy, and enhancement of both innate and adaptive immune responses. These therapeutic effects are largely mediated through the regulation of several pivotal signalling pathways, such as PI3K/AKT/mTOR, Wnt/β-catenin, JAK/STAT, and TGF-β/Smad. Moreover, APS contribute to tumour microenvironment reprogramming by promoting M1 macrophage polarisation, reducing Treg-mediated immunosuppression, and enhancing antitumour immune activity. Their synergistic application with chemotherapeutics and interventional treatments, such as transarterial chemoembolisation, has shown the potential to enhance efficacy and reduce adverse effects.

Despite the encouraging preclinical evidence, several limitations currently hinder the clinical translation of APS. Most existing studies are confined to in vitro and animal models, with limited validation in large-scale, randomised controlled clinical trials. In addition, inconsistencies in extraction methods, dosing strategies, and treatment protocols pose challenges to reproducibility and standardisation. However, a major limitation is the frequent use of concentrations above 100 μg/mL in in vitro assays, which may not be physiologically attainable in vivo due to poor bioavailability and rapid systemic clearance. Notably, several studies reviewed here (e.g., [36,37,57]) reported significant anticancer activity at or below 100 μg/mL, suggesting that lower, clinically relevant doses, especially in combination therapies, warrant further investigation. Furthermore, relatively low bioavailability and lack of well-characterised pharmacokinetic profiles of APS warrant further pharmaceutical investigation.

To enable successful translation, future research should focus on dose optimisation within achievable ranges, pharmacokinetic validation, and the development of targeted delivery systems. Rigorous preclinical models, standardised formulations, and controlled clinical trials will be essential to confirm the efficacy and safety of APS-based interventions. Future research should focus on three key areas to address these gaps. First, robust clinical trials are urgently needed to assess the safety, tolerability, and therapeutic efficacy of APS in hepatocellular carcinoma patients. Second, further mechanistic studies are required to elucidate molecular targets and identify reliable biomarkers for treatment response. Third, advancements in formulation science—such as nanoencapsulation or combination strategies—may enhance APS bioavailability and therapeutic potential, facilitating their integration into mainstream cancer care.

In conclusion, APS represent promising natural compounds with multifaceted anti-hepatocellular carcinoma properties. Their immunomodulatory and antitumour mechanisms, in conjunction with conventional therapies, support their potential role in integrative oncology. With continued scientific and clinical validation, APS may contribute meaningfully to future therapeutic strategies against liver cancer.

## Figures and Tables

**Table 1:** Major bioactive components of *Astragali* radix (Huangqi).

Category	Representative Compounds	Biological Activities	References
Polysaccharides	APS-I, APS-II, APS-A1, APS-B1, APS2-I, APS3-I	Immunomodulatory, anti-inflammatory, antiviral, hepatoprotective	[18,19,20]
Triterpenoid saponins	Astragalosides I–VIII, cycloartane, oleanane, malabaricane saponins	Cardioprotective, immunomodulatory, hepatoprotective, antitumour	[19,21,22]
Flavonoids	formononetin, calycosin, isorhamnetin, quercetin, kaempferol	Antioxidant, anti-inflammatory, antitumour, hepatoprotective	[23,24]
Amino acids	Lysine, arginine, aspartic acid, glutamic acid, proline, alanine	Nutritional supplementation, immunomodulatory	[25,26]
Phenolic compounds	Caffeic acid, ferulic acid, syringic acid, vanillic acid	Antioxidant, anti-inflammatory, hepatoprotective	[19,27]
Coumarins	umbelliferone, scopoletin, psoralen	Anti-inflammatory, antibacterial, antioxidant	[19,20]
Alkaloids	Pyrimidine and pyrrole-type alkaloids (26 types); betaine	Neuroprotective, immunomodulatory, antioxidant	[20,28]
Steroids and terpenoids	Phytosterols, monoterpenes, sesquiterpenes, tetracyclic and pentacyclic triterpenes	Anti-inflammatory, antitumour, adaptogenic	[20,29]
Minerals and trace elements	Se, Fe, Zn, Cu, Mn, Cr, Mo, Co, Cs	Essential for enzymatic functions, antioxidant, immunomodulatory	[29,30]
Fatty acids	Linoleic acid, linolenic acid, palmitic acid, oleic acid	Anti-inflammatory, cardiovascular protection	[19,31]
Other components	folic acid, ascorbic acid, quinones, inositols	General health support, metabolic balance	[20,27]

**Table 2:** Antitumour mechanisms of *Astragalus* polysaccharides in liver cancer.

Mechanism	Biological Effects	Molecular Targets/Pathways	Supporting Evidence	References
Inhibition of proliferation	Induces cell cycle arrest	↓ Cyclin D1, ↓ CDK4, ↑ p21, ↑ p53	In vitro studies on HepG2, H22 cells	[34,35]
Induction of apoptosis	Activates mitochondrial and death receptor pathways	↑ Bax, ↓ Bcl-2, ↑ caspase-3, ↑ cytochrome c	Animal models and cultured liver cancer cells	[32,47]
Regulation of autophagy	Promotes autophagic flux leading to tumour cell death	↑ LC3-II, ↑ Beclin-1, ↓ mTOR, ↓ PI3K/AKT	Autophagy markers increased in treated cells	[55,57]
Inhibition of the EMT and metastasis	Suppresses migration and invasion; reverses the EMT phenotype	↑ E-cadherin, ↓ N-cadherin, ↓ vimentin, ↓ TGF-β	EMT markers altered in APS-treated models	[58,59]
Immune modulation	Enhances innate and adaptive immune responses; reduces immunosuppression	↑ IL-2, ↑ IFN-γ, ↓ Treg, ↑ CD8^+^ T, ↑ NK cells	Tumour-bearing mouse models	[32,33,67]
Tumour microenvironment regulation	Reduces angiogenesis and hypoxia; repolarises macrophages from the M2 phenotype to the M1 phenotype	↓ VEGF, ↓ HIF-1α, ↑ iNOS, ↓ Arg-1	Improved tumour vascular structure and immune shift	[72]

## Data Availability

No new data were created or analyzed in this study. Data sharing is not applicable to this article.
